# Formative Evaluation for a Healthy Corner Store Initiative in Pitt County, North Carolina: Assessing the Rural Food Environment, Part 1

**DOI:** 10.5888/pcd10.120318

**Published:** 2013-07-18

**Authors:** Stephanie B. Jilcott Pitts, Karamie R. Bringolf, Katherine K. Lawton, Jared T. McGuirt, Elizabeth Wall-Bassett, Jo Morgan, Melissa Nelson Laska, Joseph R. Sharkey

**Affiliations:** Author Affiliations: Karamie R. Bringolf, Katherine K. Lawton, Elizabeth Wall-Bassett, East Carolina University, Greenville, North Carolina; Jared T. McGuirt, University of North Carolina at Chapel Hill, Chapel Hill, North Carolina; Jo Morgan, Pitt County Health Department, Greenville, North Carolina; Melissa Nelson Laska, University of Minnesota, Minneapolis, Minnesota; Joseph R. Sharkey, Texas A&M University, College Station, Texas.

## Abstract

**Introduction:**

Obesity prevalence in the rural United States is higher than in urban or suburban areas, perhaps as a result of the food environment. Because rural residents live farther from supermarkets than their urban- and suburban-dwelling counterparts, they may be more reliant on smaller corner stores that offer fewer healthful food items.

**Methods:**

As part of a Communities Putting Prevention to Work (CPPW) healthy corner store initiative, we reviewed audit tools in the fall of 2010 to measure the consumer food environment in eastern North Carolina and chose the NEMS-S-Rev (Nutrition Environment Measures Survey-Stores-Revised) to assess 42 food stores. During the spring and summer of 2011, 2 trained graduate assistants audited stores, achieving interrater reliability of at least 80%. NEMS-S-Rev scores of stores in rural versus urban areas were compared.

**Results:**

Overall, healthful foods were less available and of lower quality in rural areas than in urban areas. NEMS-S-Rev scores indicated that healthful foods were more likely to be available and had similar pricing and quality in rural corner stores than in urban corner stores.

**Conclusion:**

Food store audit data provided a baseline to implement and evaluate a CPPW healthy corner store initiative in Pitt County. This work serves as a case study, providing lessons learned for engaging community partners when conducting rural food store audits.

## Introduction

In the United States, obesity is a costly (1) and devastating public health problem (2). There is disproportionate obesity prevalence in rural America, particularly the rural South (3,4). The obesity disparity in rural areas versus more urban and suburban areas of the United States may be a result of the food environment, rural food deserts in particular (5–7). Rural residents may live far from supermarkets, which stock more healthful foods than do corner stores (8,9). Therefore, rural residents may be more reliant on the less-healthful foods in corner stores (also referred to as convenience stores or food marts) and similar lower-volume food venues. Corner stores and small stores in rural areas may serve an important role for “filler” shopping (ie, purchase of small quantities of groceries before a big shopping trip is needed) (10) and for residents with limited access to transportation to urban areas.

Food environments can be conceptualized in terms of the *community food environment* (defined as spatial access to food venues) and the *consumer food environment* (defined as what consumers encounter in each food venue) (11). Food store audits are one way researchers have measured the consumer food environment, and these typically entail assessment of availability, price, and quality of foods available in traditional food venues such as supermarkets and grocery stores and in nontraditional food venues such as mass merchandisers (12). A growing amount of research has assessed the consumer food environment in urban areas (13–15) as well as in rural settings, such as rural Texas (8,16,17). Two review articles (18,19) provide comprehensive summaries of tools to assess the community and consumer food environments, and many resources are available via the National Cancer Institute’s Food Environment Measures website (https://riskfactor.cancer.gov/mfe/instruments/), a continually updated online repository for food environment assessment tools. Despite these resources and assessment tools, methods for conducting food store audits in rural areas of the United States have not been assessed extensively.

Several recent US federal initiatives have sought to engage communities to promote healthful food environments, including the Healthy Food Financing Initiative and the Centers for Disease Control and Prevention’s $372.8 million Communities Putting Prevention to Work (CPPW) initiative (20). To plan for promotion of healthful foods and to evaluate the effectiveness of such efforts, high-quality data are needed, and communities must choose from among the many audit tools available to collect such data. Furthermore, few examples are available of how information gathered from food audits can be efficiently compiled and disseminated to community leaders, thus informing policy makers on what evidence supports or promotes a more healthful rural food environment.

Federal efforts to engage communities to promote healthful food environments are evolving. Assessments of the consumer food environment must meet the needs of all stakeholders working to create more healthful food environments. These include public health practitioners, researchers, policy makers, retailers, and community advocacy groups (18). Thus, in this article we describe a case study to assess the consumer food environment in rural eastern North Carolina (Pitt County) to guide planning and implementation of a CPPW healthy corner store initiative. We discuss results and lessons learned and how these may inform similar efforts to plan, evaluate, and advocate promotion of more healthful food options in rural corner stores.

## Methods

### Food stores in rural eastern North Carolina

Many efforts have been made to define and describe food deserts, which often are based on geographic proximity to the residential address, or density measures of food venue availability in specific low-income or minority neighborhoods (21). For CPPW baseline data collection, we audited a purposive sample of food stores on the basis of being either 1) located in 1 of 5 Pitt County rural food deserts (we defined a “rural food desert” as a Pitt County municipality with no chain supermarket) or 2) located in more urban municipalities that had chain supermarkets (termed “urban nonfood deserts” for our purposes), giving priority to stores located in or near low-income census block groups. (The sample was not random or representative of all food stores.) When initial results were presented to a group of Pitt County planners, also a part of the CPPW leadership team, they suggested conducting audits in corner stores in rural crossroads communities, or rural areas designated by an intersection with a small surrounding population base, not currently designated as a formal municipality. The planners suggested this approach because such communities often have a substantial number of people living nearby who may not have easily accessible fresh produce or other healthful food options. Thus, we also conducted food store audits in these small crossroads communities.

### Selection of food store audit tool

In fall 2010, we reviewed recently developed audit tools to measure the consumer food environment, published from 2005 through 2010 and found from keyword searches on Google Scholar and PubMed, focusing particularly on development, testing, and evaluation of the rural food environment. We also examined the National Cancer Institute’s Food Environment Measures website, which listed available audit tools. We worked with community partners to choose an appropriate audit tool by assessing the following factors: 1) ability to use the tool in different types of traditional and nontraditional food stores, including supermarkets, corner stores, and dollar stores, because such venues are important for rural consumers (12); 2) assessment of availability, price, and quality of food items; 3) inclusion of canned vegetables and meats, because both are frequently purchased by low-income people (22) and rural residents may live far from supermarkets that carry fresh vegetables and meats (9); 4) time required in the store to complete the audit, because we did not want to foster distrust between store owners and research assistants conducting audits; 5) ability to calculate a score for each food store assessed, because we wanted to be able to easily disseminate results to community stakeholders; and 6) applicability to varied communities and racial/ethnic groups (vs applicability to only select racial/ethnic groups), because CPPW goals focused on all racial/ethnic groups using corner stores. Because it met the criteria, the evaluation team decided to use the Nutrition Environment Measures Survey-Stores-Revised (NEMS-S-Rev) (14) as the food store audit tool most appropriate for the purposes of meeting CPPW goals.

### Using the NEMS-S-Rev to audit food stores

In spring and summer of 2011, we audited 42 food stores in 10 Pitt County municipalities and 4 designated crossroads communities of Pitt County, using the NEMS-S-Rev to measure the food environments. In urban (nonfood desert) areas, we selected a purposive sample of stores considering geographic variability (in location of stores) and store proximity to low-income areas. We included corner stores and chain supermarkets, as described by the NEMS-S-Rev protocol (14). Because CPPW focused on making changes to corner stores, we audited 33 corner stores. For comparison, we also audited 9 chain supermarkets. For the purposes of this article, we defined corner stores as both convenience stores and food marts (Standard Industry Classification code 541101–5). Convenience stores were defined as venues selling limited amounts of a medium variety of canned goods, dairy products, prepackaged meats, and other grocery items. Food marts were defined as similar to convenience stores in terms of the size and variety of items they sell, but they are associated with a gas station. At the time the study was conducted, there were 20 supermarkets and 65 corner stores in Pitt County.

Stores were scored on availability, price, and quality in each of 12 food categories (milk, cheese, fresh fruit, fresh vegetables, frozen and canned vegetables, meat, meat alternatives, beverages, bread, grains, cereal, and chips). Availability was defined by whether certain food items were available in the stores, with more points assigned to stores with a greater number of healthful food items available. The range of possible availability scores was 0 to 34, with higher scores indicating that the store had more healthful foods available than stores with lower availability scores. The price score was measured by comparing the price of the more-healthful option (eg, whole-wheat bread) to the price of the less-healthful option (eg, white bread). If the more-healthful option had a lower price than the less-healthful option, the store received 2 points; if the prices were the same, the store received 1 point; and if the more-healthful option had a higher price than the less-healthful option, the store lost 1 point. The range of possible price scores was −12 to 24; higher scores indicated that more-healthful options cost less than less-healthful options or that less-healthful options were more expensive than more-healthful options. Negative scores were possible, because stores received negative points for having more-healthful items priced higher than less-healthful items. Quality was measured only for fruits and vegetables and was a subjective measurement. Graduate assistants determined produce quality by assessing bruising, discoloration, and rotting. One point was assigned if 25% to 49% of the fruits and vegetables were acceptable, 2 points if 50% to 74% were acceptable, and 3 points if 75% or more were acceptable. The quality score for fresh fruits and vegetables could range from 0 to 6, with higher scores indicating higher quality. According to standard NEMS-S-Rev protocol, the overall score was calculated by summing availability, pricing, and quality scores (possible overall scores ranged from −12 to 64).

Before assessing stores for baseline data collection and scoring, 2 research assistants independently and simultaneously audited 1 of each store type including a chain supermarket, small grocery store, convenience/corner store, and dollar store. To increase data collection credibility and interrater reliability, and to settle discrepancies, the 2 graduate assistants jointly compared results on the independently scored stores. Then, for each food store included in the final purposive sample, the graduate assistants assessed stores individually then jointly to compare assessments, resolve discrepancies, measure interrater reliability, and score stores. A final interrater reliability of 80% or greater was achieved for all food store audits. We did not conduct significance tests because of the small purposive sample of stores selected.

## Results

Overall, stores in rural Pitt County had lower mean NEMS-S-Rev scores than did the stores in more urban areas, which, by definition, included supermarkets (Table). However, supermarkets in urban areas did not seem to have healthful options as competitively priced as did corner stores in urban and rural areas, as indicated by the negative NEMS-S-Rev price score for supermarkets.

**Table:** Nutrition Environment Scores^a^ of Food Stores in Rural Food Desert and Urban Municipalities in Pitt County, North Carolina (N = 42)

Type of Store	Rural Food Desert Stores (n = 17)	Urban Nonfood Desert Stores (n = 25)
**Supermarket (n = 9), mean (SD)**	NA	n = 9
Availability	NA	29.7 (3.7)
Price	NA	−2.1 (2.0)
Quality	NA	5.2 (0.67)
Overall	NA	32.8 (4.3)
**All corner stores (n = 33), mean (SD)**	n = 17	n = 16
Availability	9.1 (3.0)	8.4 (2.6)
Price	2.3 (1.6)	2.4 (1.5)
Quality	0.06 (0.24)	0.06 (0.25)
Overall	11.4 (3.0)	10.8 (3.0)
**Corner stores^b^ (n = 25), mean (SD)**	n = 9	n = 16
Availability	7.7 (3.1)	8.4 (2.6)
Price	2.4 (1.7)	2.3 (1.5)
Quality	0.1 (0.33)	0.06 (0.25)
Overall	10.2 (3.5)	10.8 (3.0)

Abbreviation: SD, standard deviation; NA, not applicable.

a The nutrition environment was assessed using the Nutrition Environment Measures Survey-Stores-Revised (14).

b Excluding those in crossroads communities, or rural areas designated by an intersection with a small surrounding population base, not currently designated as a formal municipality.

Among corner stores, the focus of the Pitt County CPPW initiative, those in rural areas (n = 17) had higher availability scores but similar price and quality scores than corner stores in urban areas (n =16). The availability and overall NEMS-S-Rev scores were slightly lower for corner stores in rural areas when corner stores in crossroads communities were excluded from the analysis.

## Discussion

Audited supermarkets had a price score of −2.1 and corner stores had a price score of 2.3, indicating that more-healthful items were more competitively priced in the corner stores audited than in the audited supermarkets. However, as expected, supermarkets had higher overall NEMS-S-Rev scores than corner stores. At corner stores, specifically, those in rural areas had higher NEMS-S-Rev availability scores than did corner stores in more urban areas, which suggests that corner stores in rural areas had more healthful options available than corner stores in urban areas. These results provided a baseline from which to implement and evaluate a CPPW healthy corner store initiative in Pitt County.

Our study has limitations. We used a small, nonrandom, purposive sample of food stores. Because we did not audit a representative or random sample of stores, audit results may not represent the overall patterns of availability, pricing, and quality of foods in Pitt County. However, we did purposively sample stores, ensuring that stores audited were frequented by rural and low-income customers, to ensure that CPPW efforts reach those most at risk for obesity. Also, because audit tools often measure only select food items, they may not capture the rural consumer food environment in its entirety. Analyses of national data related to rural consumers’ food choices can inform development of future audit tools to more accurately capture the rural consumer food environment. We were not able to assess certain aspects of stores (eg, product placement, whether the store sold food from a grill, presence of advertisements of unhealthful foods). 

Strengths of our efforts include assessment of the availability, price, and quality of healthful versus unhealthful food items, using a validated audit tool. We evaluated appropriate audit tools based on available literature and selected tools to gather credible evidence on the basis of time, cost, and technical demand. We used a collaborative and tailored approach to engage community partners in the planning and evaluation of the CPPW healthy corner store initiative.

Our food store audit results were compiled and presented to the CPPW team to inform planning and evaluation of the healthy corner store initiative. NEMS-S-Rev scores were used to determine changes to make in corner stores and were used as an evaluation tool after healthful changes were made. Based on store owner or manager interest in partnering with the CPPW initiative, we selected 11 stores in which to conduct qualitative interviews with food store owners and managers, as reported in Part 2 of this evaluation report and as done by others (23,24) to determine relevant characteristics of the store’s customer base and customer shopping patterns and to learn about inventory decisions. Finally, we conducted baseline customer intercept surveys in 9 of the 11 selected corner stores to learn more about customer shopping patterns and healthful food options customers would purchase from corner stores, also reported in Part 2 of our formative evaluation report. We compiled tailored corner store reports for each of the 11 stores, and hand delivered the reports to stores with a letter thanking them for their participation. These reports and letters were used to build rapport with local store owners. Reports included aggregated and individualized NEMS-S-Rev results, as well as results of the store owner qualitative interviews and the customer surveys, and we distributed these to the CPPW project team as well as to each store owner.

When assessing the consumer food environment of small stores, store owners and managers may perceive the people conducting audits as competitors or fear that audit results will have negative repercussions. Therefore, auditor intentions must be clearly presented to store owners. Rapport with store owners and managers can be built during food audits, if the process is presented in a nonthreatening way. Although all audits for this study were conducted during daylight hours, the safety of auditors was occasionally an issue in corner stores. This issue can be resolved by partnership with community agencies that have connections to local law enforcement resources. In addition, as the NEMS-S-Rev did not include detailed information to assess sodium content of foods, as new nutrition recommendations emerge, such as those regarding sodium, audit tools should include some assessment of availability of low-sodium options. Finally, as recently suggested by Rose et al (25), because the NEMS-S-Rev does not include any assessment of the broader community food environment, a more complete picture of the rural community and consumer food environment could be provided by combining measures of food store access using geographic information systems, with measures of availability, pricing, and quality of healthful items as assessed using food store audit tools.

To aid the CPPW team in identifying which store to pilot the healthy corner store initiative, we also compiled a list ranking stores in terms of customer volume, location of the store (proximal to low-income areas), and willingness of the owner to provide more-healthful food in the store. The combination of these efforts, described here and in Part 2 of this report, as well as reports of previous efforts (23,24) guided CPPW planning for effectively promoting healthful foods in both rural and urban food stores.

There is a need to ensure that low-income residents, particularly those in the rural South, have access to healthful foods. Many US communities have been funded through the CPPW initiative to address the need for access to more-healthful food via environmental and policy changes (20). In Pitt County, as has been done in other communities across the nation, we collaborated with corner store owners to supply and promote more healthful food choices in rural and underserved areas, and to support corner stores as economically thriving and sustainable rural food venues. We hope that the Pitt County experience will offer guidance to others collecting empirical data to pursue policy and environmental changes in the rural food environment.
